# Medical Clinic of Atlanta Medical College

**Published:** 1877-01

**Authors:** J. G. Westmoreland


### MEDICAL CLINIC OF ATLANTA MEDICAL COLLEGE.

BY J. G. WESTMORELAND, M.D.

*Oct.* 23, 1876.—To-day, gentlemen, we have presented for treatment this child, fourteen months old, which shows decided febrile symptoms, with swollen condition of the gums. The history, as obtained from the mother, gives us the further information that diarrhœal discharges are frequent and copious. In this case the first dentition has been unusually delayed. Generally teething has advanced as far at seven months as is found in this case at fourteen. Such disturbance as this presents is of frequent occurrence during the warm season. From the last of May to the first of September is a dangerous season for teething children. So late, however, as the present we rarely meet with the serious bowel trouble exhibited in this child. It will be observed, however, that according to the mother's statement, evidences of enteric irritation were manifested several months since, and which were doubtless the result of the teething process. The gummy structure, through which the teeth must pass in making their appearonce, is sometimes tough and unyielding; so that, for a long time, there exists a constant source of irritation—the sharp point of the tooth pressing against the gums. We sometimes find the teething delayed by general bad health and imperfect nutrition, but from the child's appearance, it is not probable that such cause has existed in this case. It is more likely that this, like many others, was naturally late in commencing dentition; and, like some others, has unusual toughness of the gums. All the children of some mothers have their teething delayed to the age of ten or twelve months, while those of other women as uniformly commence at the age of six months, and some even earlier.

This irritation of the gums by the protruding teeth is the source of various and serious lesions, particularly in the alimentary canal, and sometimes in the brain. The disease is known as "summer complaint," cholera infantum, diarrhœa, etc., most frequently attacks teething children, and is generally the result of this process. As stated, the warm season is more prolific of this disturbance, and often those tenacious of life, having strong vital powers, hold up under the disease for weeks and even months, being saved finally by the timely aid of cold weather.

Continued high temperature of the atmosphere naturally tends to develop enteric irritation, engorgement, and inflammation, in persons of all ages, and hence the reflex impression from the inflamed gums is more likely to develop serious lesion of the alimentary mucous membrane. Sluggish portal circulation, consequent upon excessive heat, and leading to congestion of the abdominal viscera, may be mentioned as an explanation of the general tendency to bowel affections in summer.

Now, gentlemen, in view of all these facts, what are the indications in the case before us, and how are they to be filled? Two prominent objects present themselves, both of which must be accomplished in order to the cure of this case, viz.: the control of enteric inflammation and relief of irritation in the gums. It may be thought sufficient to relieve the gums from the constant jagging and pressure of the teeth, since it is probably the cause of trouble; or that the bowel lesion alone be relieved. Now, while it is true that in some instances, before the inflammatory condition in the mucous membrane of the bowels has become thoroughly established and fixed, removal of the prime cause may result very soon in arrest of the bowel trouble. We cannot, however, in this case, confidently expect prompt relief from the diarrhœal symptoms without the use of remedies for the direct accomplishment of this object. Neither have we any assurance of permanent relief from the enteric irritation, until the alveolar excitement has abated; and even after this has been done, the enteric inflammation having been firmly established, does not subside always without remedies directed specially to this part.

Finally, the means by which these indications are to be filled become the great practical question. And first, we suggest relief to the swollen gums, and propose at once to divide them by a thorough incision down to the teeth. Thus this prolific source of trouble will be removed by hemorrhage from the inflamed gums and freedom from the irritation of contact with the teeth. The cut surfaces speedily unite very often, and necessitates a repetition of the operation, particularly when the teeth are yet deep within the gums, affording no means of keeping the edges separated. Even when the portion divided is very thin, slow growth of the teeth, from general debility, may allow reunion to occur.

The next, and as you may conclude, the most essential step in the treatment of the case, consists of means addressed directly to the bowels. In this course success depends upon the observance of certain therapeutic principles. Two of these are, rest or quietude, as of any inflamed part, and freedom from irritating causes. These are insisted on by the surgeon, and are no less essential in the treatment of ordinary diseases. I therefore prescribe fluid, unirritating articles of food, that will leave no embarrassing contents in the bowels, and opium to allay the excessive peristaltic movement. Opium arrests, not only the increased regular and spasmodic contractions of the muscular coat, but the irritation existing in the mucous membrane, also.

Much has been said and written, pro and con, of astringents in the treatment of acute inflammation. As you know, they contract and make firm and solid the soft tissues with which they come in contact. In this action, then, the congestion of parts, upon which inflammation mainly depends, is lessened. Of this action we therefore desire to avail ourselves in the treatment of this case.

Protection and soothing influence, if it be possible to afford them, are desirable. Mechanical means, such as demulcent or mucilaginous substances, are resorted to for these purposes, and I think with decided benefit.

As before stated, sluggish portal circulation tends to the damming up or engorgement of the abdominal viscera, and amongst them the bowels. The liver, after unusual excitement and activity, during the warm season, becomes to some extent torpid. Engorgement, the result of this, interferes with the circulation through the portal vessels which carry the blood from the bowels, and hence the enteric congestion.

Now, to meet this very commonly existing difficulty, and one which is sometimes the prime cause of bowel affections, some preparation of mercury is required in the commencing treatment. In the management of enteric inflammation, as well as that of other parts, counterirritation is often indispensible. Whether the disease pervades the whole canal, or is confined to the large or small intestines, blisters, in obstinate cases, become the "sheet anchor" of our hope.

In accordance with these principles, I make for the case the following prescriptions:

℞.—Calomel, . . . . . . . . gr. iij

Syrup, . . . . . . . . . f℥ss

Mix.

S. Give at once.

℞.—Tinct. Opii Camphorata, . . . . . f℥ij

Tinct. Catechu, . . . . . . . f℥ij

Acacia Mucilage, . . . . . . f℥iij

Mix.

S. One teaspoonful three times daily, or more frequently, if required to restrain the evacuations.

The gums having been well cut down to the teeth, these prescriptions are considered sufficient to relieve this case, without a blister or other counter irritant.

It will be observed that the dose of calomel is large for a child of fourteen months. Three grains in this case is about equivalent to thirty-five grains for an adult. You should remember, however, that children require, of mercurial preparations, a larger proportion of the adult dose, than of other remedies. Again, experience, I think, justifies the opinion that minute doses of calomel, frequently repeated, tend to aggravate moco-enteric irritation, and hence I have long since abandoned the old practice of one-eighth grain of calomel every two hours in cholera infantum, or summer complaint in children.

